# Upregulation of microRNA-451 attenuates myocardial I/R injury by suppressing HMGB1

**DOI:** 10.1371/journal.pone.0235614

**Published:** 2020-07-17

**Authors:** Jianlei Cao, Yurong Da, Hang Li, Yuanyuan Peng, Xiaorong Hu

**Affiliations:** 1 Department of Cardiology, Zhongnan Hospital, Wuhan University, Wuhan, P.R. China; 2 School of Medicine, Jianghan University, Wuhan, P.R. China; Chinese Academy of Medical Sciences and Peking Union Medical College, CHINA

## Abstract

Both MicroRNAs and HMGB1 took part in pathological process of myocardial I/R injury though several signaling pathways. We hypothesized that mircoRNA451 (miR-451), a group of small non-coding RNAs, could improve this injury by inhibiting HMGB1. Male SD rats were randomly distributed into 5 groups and subjected to I/R process. After 24 hours of reperfusion injury, the serum content of CK and LDH, the content of MDA in tissue and activity of SOD were detected; The infarcted areas were defined by TTC staining and Evans Blue; TUNEL staining and cleaved-Caspase 3 were used to test apoptosis; HMGB1 was detected by real-time fluorescence quantitative PCR and Western Blotting. Compared with the I/R and I/R+Ad-GFP group, upregulation of miR-451 could reduce the infarcted areas, cardiomyocytes apoptosis index, expression of cleaved-caspase 3 and content of CK and LDH significantly(P<0.05); Meanwhile, upregulation of miR-451 could also obviously inhibit HMGB1, the increase of MDA and the decrease of SOD (P<0.05). So this study revealed that upregulation of miR-451 could prevent myocardial I/R injury by suppressing HMGB1.

## Introduction

Cardiovascular disease is a major cause of human death. Myocardial Ischemia Reperfusion Injury (MIRI) can cause heart failure, re-infarction, arrhythmia and other complications, which pose a serious threat to the life and health of patients [1, 2]. MIRI often occurs after myocardial infarction and cardiac bypass surgery [3]. MIRI involves a series of complex pathological mechanisms, including oxidative stress, inflammatory response, cardiac dysfunction, calcium pump dysfunction, myocardial cell necrosis and apoptosis, which is a major obstacle in the treatment of ischemic heart disease [2].

MicroRNAs, containing 18 to 25 nucleotides, are endogenous noncoding RNAs, which participate in post-transcriptional regulation of protein expression, lead to the target mRNAs translational degradation or repression [4, 5]. Increasing evidences have showed that miRNAs participate in the regulation of cardiac pathological processes, however, the expression profiles and targeting proteins of miRNAs are altered distinctly during these diseases [6]. More recently, several miRNAs have been found to play an important role in the process of I/R injury [7–9]. It has been known that miR-1, miR-133, miR-320 and miR-451 may undergo significant changes in response to oxidative stress and inflammatory, which further regulated the cardiomyocyte apoptosis through targeting molecules [10–13].

HMGB1 is a non-histone nucleoprotein containing two lysine-rich DNA binding regions and an unusual C-terminal acid tail. It consists mainly of aspartic acid and glutamate residues and is released passively by apoptotic, necrotic and innate immune cells. Previous studies have found that HMGB1 can promote autophagy and inflammation [14]. HMGB1 can activate natural immunity by way of signal transduction in the RAGE and TLRs, mediate inflammation, tissue injury and cytokine release [15]. Our previous studies have shown that inhibited the expression of HMGB1 by various small molecule drugs could inhibit inflammation process and prevent against myocardial I/R injury [16, 17]. However, the protected effect induced by interaction between miRNAs and HMGB1 remains unclear.

Recent studies suggested that miR-451 was downregulated in ex and vivo I/R rat hearts significantly, which further involved in the prevention of cell death, simulated I/R-triggered in human cardiomyocyte [18, 19]. Meanwhile, we use TargetScan software to confirm that HMGB1 is a potential action site for miR-451. However, each miRNA has more than 1,000 target genes, every gene has been regulated by many different types of miRNAs [6], the relationship between miR-451 and HMGB1 has not been explored before. In this study, we attempt to demonstrate whether miR-451 induce cardioprotection in I/R injury by attenuating HMGB1 induced pro-inflammatory effect.

## Materials and methods

### Construction of adenoviruses

An sequence containing pre-miR-451 and asmiR-451 was constructed and amplified by PCR using the following primers: After cloned into an pDC316- siRNA shuttle vector and co-transfected HEK393 cells with pBHG lox ΔE1,3 Cre vector in an Ad-Max system, we harvested the recombinant adenovirus containing pre-miR-451(5’-TTTGGGAATGGCGAGGAAACCGTTACCATTACTGAGTTTAGTAATGGTAATGGTTCTCTTGCTGCTCCCACA-3’) and asmiR-451(Forward primer: 5’-CCGGAACTCAGTAATGGTAACGGTTTTTTTTG-3’. Reverse primer: 5’-AATTCAAAAAAAACCGTTACCATTACTGAGTT-3’). Recombinant adenoviruses were propagated on HEK293 cells and purified by using Adeno-X^™^ system as previously described.

### I/R injury model

All experiments conformed to the Guide for the Care and Use of Laboratory Animals published by the US National Institutes of Health. The protocol was approved by the Animal Ethics Committee of Wuhan University. Male SD (200-250g) were divided into five groups randomly:

1) Sham group(SO, N = 10): Rats underwent surgical thoracotomy to expose the heart and needle insertion below the anterior descending branch, but did not induce ischemia; 2) Ischemia reperfusion injury group (I/R,N = 15): Three days before surgery, PBS(150μl) was injected into 6 different anterior wall of rat left ventricle, then the left anterior descending branch was occlused for 30 minutes and reperfused for 24 hours; 3) Ad-GFP and I/R group(N = 15): Rats were injected Ad-GFP (150μl, 1*10^10^ pfu/rat) and other steps are the same as I/R; 4) Ad-miR-451 and I/R group(N = 15): Rats were injected Ad-miR-451 (150μl, 1*10^10^ pfu/rat) and other steps are the same as I/R; 5) Ad-asmiR-451 and I/R group(N = 15): rats were injected Ad-asmiR-451 (150μl, 1*10^10^ pfu/rat) and other steps are the same as I/R.

Rats were used sodium pentobarbital (40mg/kg) to anesthetize by Intraperitoneal injection. Mechanical ventilation was via tracheal intubation, Cut the skin, bluntly separate through the third intercostal space to expose the precordial area, inelastic 6–0 thread was passed under it 2mm below the left atrial appendage. Then, a small section of silicone tube is placed over the vessel and ligated with the vessel to occlude the LAD. Then chest cage was closed in layers for 30min. For reperfusion, the knot was released for a 24h. The success of I/R model was verified by visual measurement of myocardial tissue below ligation point color change and electrocardiogram monitoring. For ischemia period, the ST-segment elevated in Leads-II and ischemia region turned into cyanosis on surface. For the reperfusion, the elevated ST segment set down and the surface of ischemia region turned back red.

### Triphenyl tetrazolium chloride staining

After 24h reperfusion, re-anesthetize rats and open the thoracic cavity to expose the anterior cardiac region, then LAD was reoccluded at the same site. External jugular vein was injected by 1mL of 1% Evans blue dye to identify the location that was not supplied by LAD. To determine the infarct size of the heart, ventricular slices (2 mm) were incubated in 1% TTC (10 minutes, 37 °C), the TTC unstained area is the infarct area. Infarct size is expressed as a percentage of IAR (% IAR).

### TUNEL staining

Apoptosis in heart was measured by TUNEL staining. The apoptosis index was determined by randomly counting TUNEL-positive myocytes in 10 fields.

### Real-time quantitative PCR

Total RNA of cardiac tissues was extracted by Trizol reagent (Roche, USA). For the quantification of miR-451, RNA was reverse transcribed into corresponding cDNA with oligo(dt) from total RNA. HMGB1 expression and miR-451 content were measured by RT-PCR analysis using a SYBR Green -based qPCR kit (Roche, USA).

### Western blotting

As mentioned before, quantitative immunoblotting was performed with HMGB1 and cleaved caspase 3 antibodies [16]. GAPDH antibody (1:5000 dilution; cell signaling) was used as the loading control.

### Assay of CK and LDH

The injury of myocardium was evaluated by the serum concentration of CK and LDH. Protocols accorded to the instructions of manufacturer. (JianCheng Bioengineering Institute, Nanjing, China).

### Assay of SOD activity and MDA

The lipid superoxide level and indexes of oxygen free radical in cardiac tissue represent oxidative stress damage. MDA and SOD were tested followed the protocol.

### Statistical analysis

SPSS 17.0 was performed. All data are expressed as mean ± standard deviation (SD). One-way analysis of variance (ANOVA) with Sidak correction was applied for multiple comparisons. p<0.05was defined statistical significance.

## Results

### Infarcted areas

**Fig 1:**
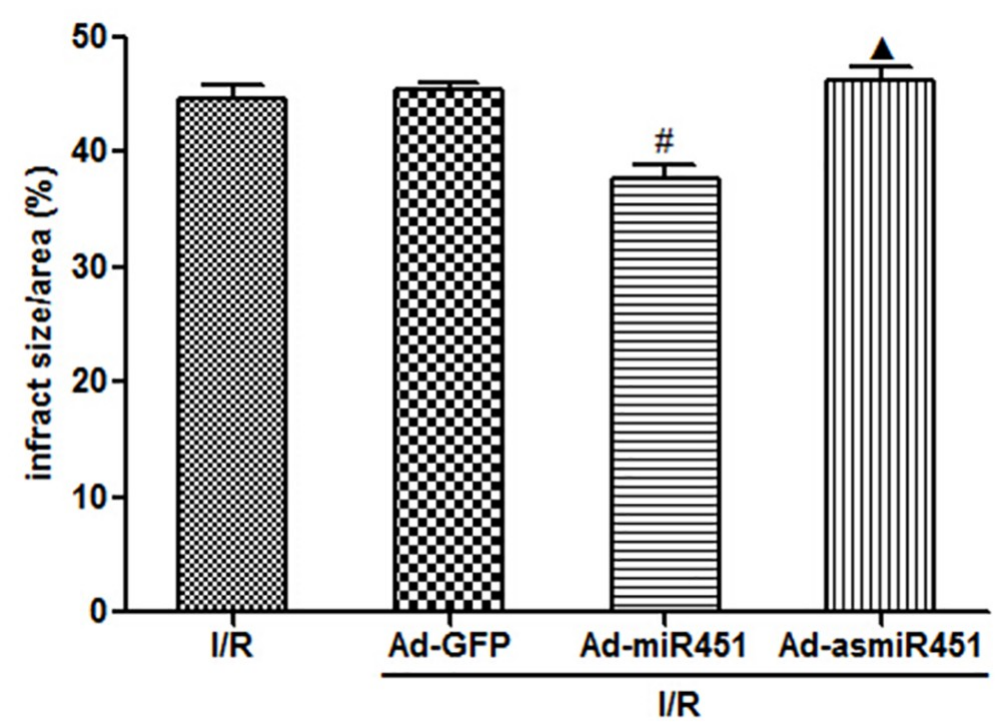
Effect of miR-451 on infarcted area in I/R. #: P<0.05 compare with I/R group; ▲: P>0.05 compare with I/R group.

24 h reperfusion later, treatment with Ad-miR-451 could decrease the infarcted size caused by I/R compared with that in I/R group and I/R+ Ad-GFP group (P<0.05). Nevertheless, compared with I/R group and I/R+ Ad-GFP group, the infarcted area was not increased in Ad-asmiR-451+I/R group (Fig 1).

### CK and LDH contents

**Fig 2:**
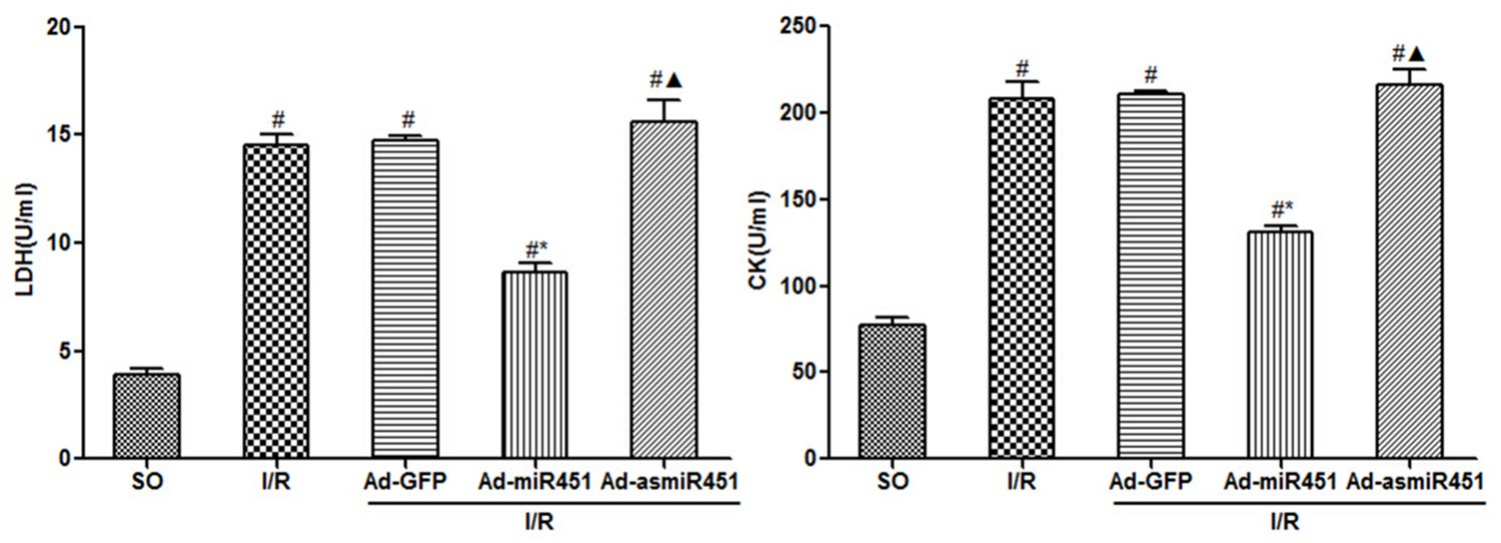
Effect of miR-451 on CK and LDH in I/R. #: vs P<0.05 compare with SO group; *: P<0.05 compare with I/R group; ▲: P>0.05 compare with I/R group.

24h after reperfusion, compared with SO group, CK and LDH contents in I/R group and I/R+ Ad-GFP group increased significantly (P<0.05). The intervention of Ad-miR-451 significantly inhibited in CK and LDH contents caused by reperfusion injury (P<0.05). Whereas, compared with I/R group and I/R+ Ad-GFP group, there was no significant difference in the content of CK and LDH in I/R+Ad-asmiR-451 group (Fig 2).

### MDA and SOD level

**Fig 3:**
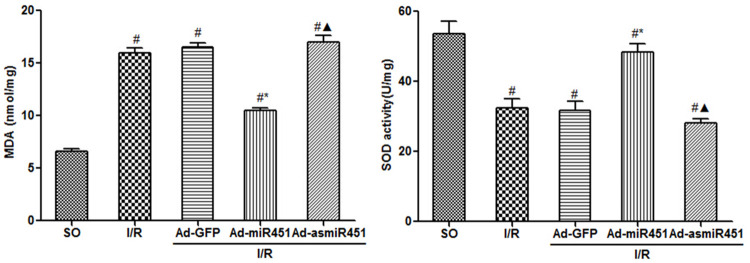
Effect of miR-451 on MDA and SOD in I/R. #: vs P<0.05 compare with SO group; *: P<0.05 compare with I/R group; ▲: P>0.05 compare with I/R group.

Compared with the SO group, MDA levels were significantly increased in the I/R and I/R+Ad-GFP groups at 24 h after reperfusion, while SOD levels were significantly lower (P<0.05). Treatment with Ad-miR-451 could significantly inhibit this change. (both P<0.05). However, there was no difference for MDA and SOD levels in the I/R+Ad-asmiR-451 group compared with the I/R and I/R+Ad-GFP groups (Fig 3).

### Effect of mir-451 on myocardial cell apoptosis caused by reperfusion injury

**Fig 4:**
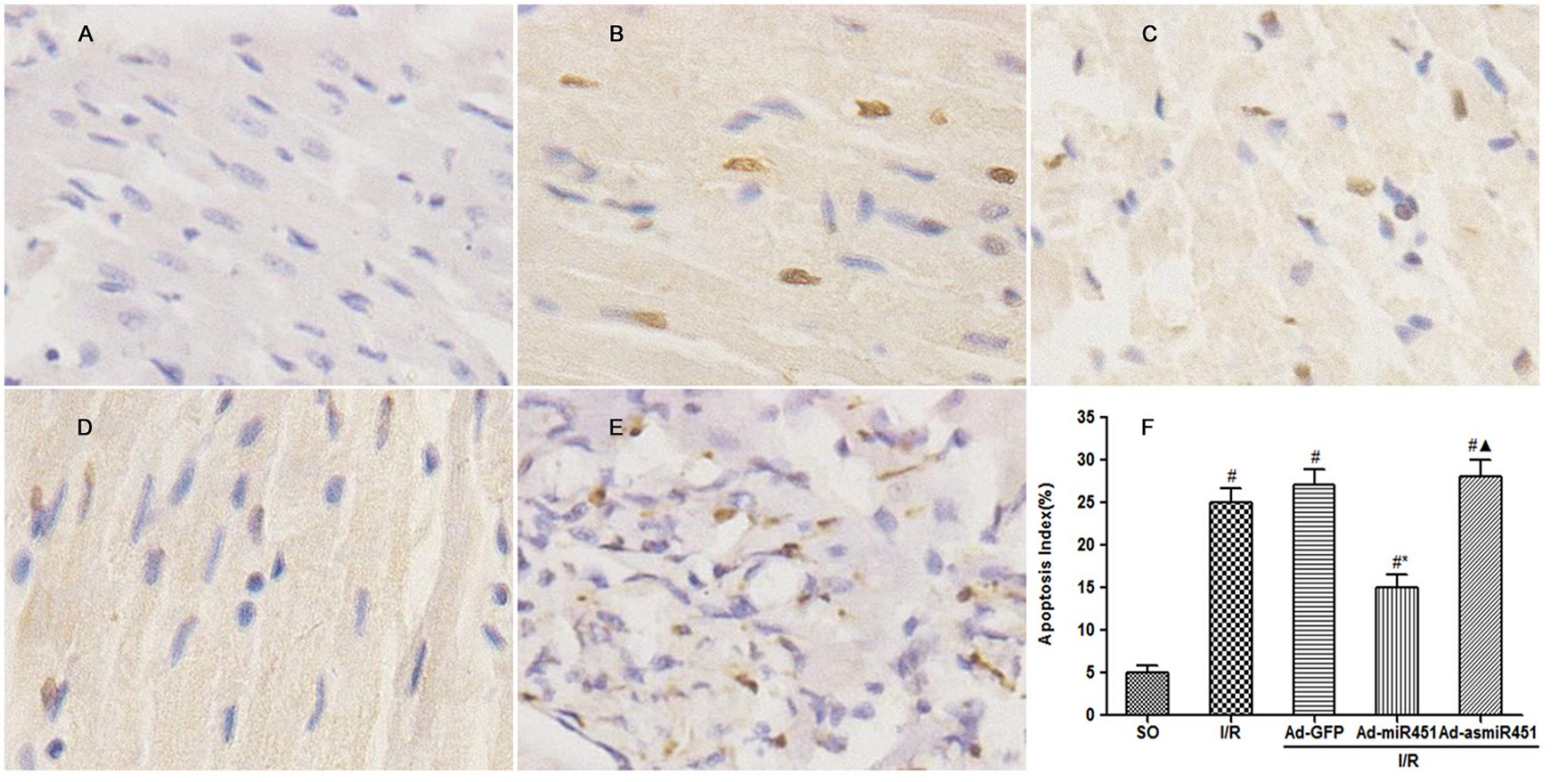
Effect of miR-451 on cardiomyocytes apoptosis. (A-E) Results of cardiomyocyte apoptosis in each group assessed by TUNEL staining. (F) The index of apoptosis was detected by counting TUNEL-positive myocytes randomly in 10 fields. #: vs P<0.05 compare with SO group; *: P<0.05 compare with I/R group; ▲: P>0.05 compare with I/R group.

**Fig 5:**
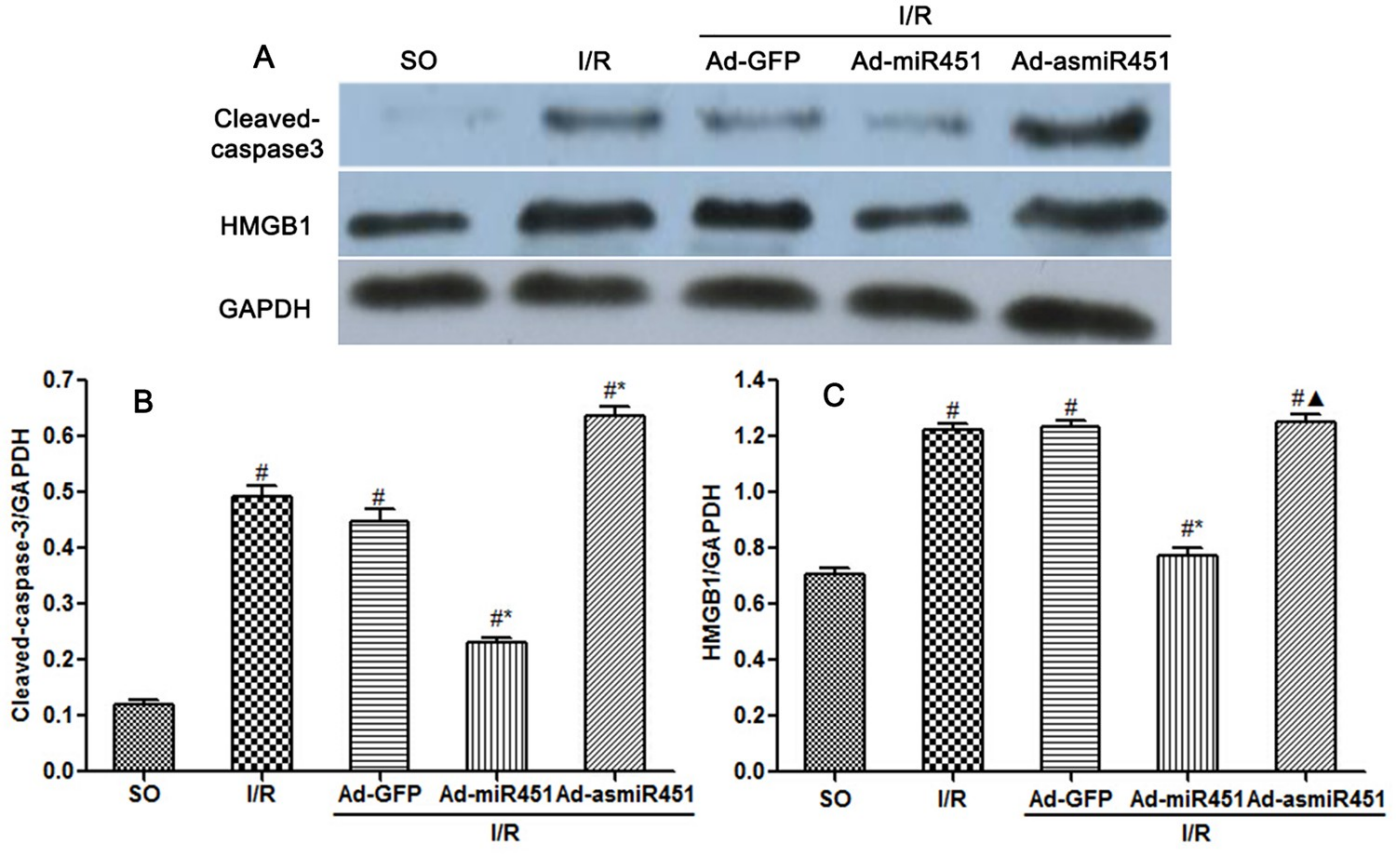
The protein expression of cleaved-caspase 3 and HMGB1. (A) The relative expression of HMGB1 protein and cleaved-caspase 3 protein. (B) Effects of miR-451 on cleaved-caspase3 expression. (C) Effects of miR-451 on HMGB1 expression. #: vs P<0.05 compare with SO group; *: P<0.05 compare with I/R group; ▲: P>0.05 compare with I/R group.

Cardiomyocytes apoptosis was determined by the TUNEL assay and the protein expression level of cleaved- caspase3 contained in myocardium. Ad-miR-451 decreased I/R-induced cardiomyocytes apoptosis and the expression level of cleaved-caspase3 (P<0.05). However, Ad-asmiR-451 failed to increase I/R-induced cardiomyocytes apoptosis but resulted in increased expression level of cleaved- caspase3 (P<0.05) (Figs 4 and 5).

### The effect of miR-451 on the expression of HMGB1 in I/R induced myocardium

**Fig 6:**
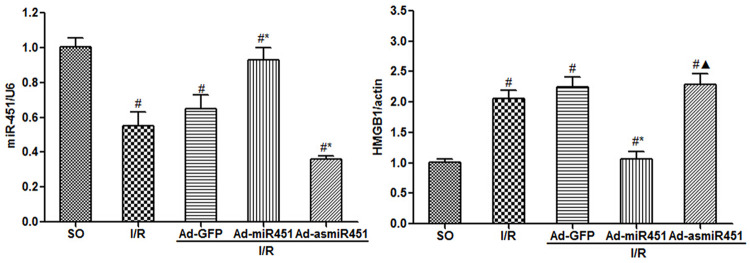
The effect of miR-451 on the expression of HMGB1. (A) The level of miR-451 in each group. (B) Effect of miR-451 on the expression of HMGB1 mRNA in cardiomyocytes. #: vs P<0.05 compare with SO group; *: P<0.05 compare with I/R group; ▲: P>0.05 compare with I/R group.

HMGB1 expression in myocardium was assessed by qRT-PCR and western blotting. QRT-PCR revealed that the concentration of miR-451 was significantly increased and HMGB1 mRNA was obviously lower in Ad-miR-451 group compared with in I/R or I/R+Ad-GFP group (P<0.05). Western blotting analysis suggested that the HMGB1 protein level had similar results with qRT-PCR. However, compared with in I/R and I/R+ Ad-GFP group, the HMGB1 mRNA and protein levels were not altered obviously in I/R+Ad-asmiR-451 group (Figs 5 and 6).

## Discussion

In this study, we have attempted to identify the regulation mechanism of HMGB1 in the field of miRNAs. We used in vivo models to show that miR-451 attenuated the inflammatory status, oxidant stress injury and cell apoptosis. The results of this study showed that miR-451 expression decreases in I/R, meanwhile HMGB1 expression increased (Fig 6). The expression of HMGB1 was significantly reduced after up-regulation of miR-451 in myocardial tissue (Figs 5 and 6). At the same time, up-regulating miR-451 reduces the area of myocardial infarction (Fig 1), relieves myocardial injury (Fig 2), and reduces myocardial cell apoptosis (Fig 4). In addition, in previous experiments, we used bioinformatics algorithm analysis to predict the miR-451 binding site at the 3′-UTR recognition of HMGB1, and further verified the relationship between miR-451 and HMGB1 by luciferase experiments [20]. In conclusion, for the first time, our results showed a cardioprotective effective of miR-451 during IR injury, which was associated with the downregulation of HMGB1.

HMGB1 is known as an early mediator of organ damage and inflammation during myocardium I/R injury [14, 21]. Consistent with previous studies [16, 17], this study discovered that HMGB1 level was obviously increased in myocardial I/R injury. What’s more, HMGB1 could facilitate the secretion of other inflammation cytokines, which induced neutrophil infiltration and apoptosis that further aggravated this pathological process [22]. Our work and that of others have shown that some drugs including minocycline, asperosaponin X and ethyl pyruvate could alleviate myocardial I/R injury by down-regulating the expression of HMGB1, which further modulated inflammatory signalling pathways including MAPK and NF-ΚB signaling pathway [15–17, 23]. Moreover, the pre-treatment with HMGB1 could activate the PI3K/Akt signalling pathway and on the meantime other reperfusion injury salvage kinase pathways like mitochondrial potassium channel, protein kinase C, and endogenous nitric oxide synthase, which further induced cardioprotecion during I/R [24–26].

Increasing evidences suggested that miRNAs play crucial roles in various heart diseases by degradation or translational inhibition of their target genes. Several ischemia and reperfusion related miRNAs including miR-320, miR-29, the muscle-specific miR-1, miR-133 and miR-21 as well as their target genes including HSP60, HSP 70, Bcl-2, HSP 20, and PDCD4 have been identified and validated [7, 11, 27]. These miRNAs represent collaborating or opposing effects during this process. Noteworthy, miR-451 was downregulated in ex vivo I/R models significantly, which further identified as cardioprotective miR via targeting CUGBP2-COX-2 pathway [19]. Moreover, miR-451 could modulate the oxidant stress injury by targeting Rac-1 during the precondition of I/R injury [28]. However, in our study, we first identified predictive miR-451-binding sites at 3’-UTR of HMGB1 through bioinformatics algorithm analysis, and further verified the relationship between microRNA-451 and HMGB1 through luciferase assay. Meanwhile, the overexpression of miR-451 was correlated with the significantly downregulated protein level of HMGB1. Moreover, the overexpression of miR-451 reduction of the level of inflammatory factor, ROS productions and cardiomyocyte apoptosis. All these effects were blocked by repression of miR-451, suggested that mir-451 plays an important role in the cardioprotection of HMGB1 against ischemia-reperfusion injury.

There are some limitations in this study. We did not measure the different regions (ischemic zone, border zone, and remote zone) of the heart separately, which may result in different results due to different infarct areas of different samples. At present, there are few studies on the effect of directly inhibiting or increasing HMGB1 on the infarct size, which may become a potential research direction.

## Conclusions

In general, our results confirm that miR-451 plays a significant role in improving myocardial I/R injury by inhibiting HMGB1. Although this relationship is one of the mechanisms underlying miR-451 protected against ischemia and oxidative stress, collective evidences suggested that miR-451 may act as a potential drug for myocardium reperfusion injury.

## Supporting information

S1 File (RAR)
